# Serological and molecular epidemiology of the Dengue, Zika and Chikungunya viruses in a risk area in Brazil

**DOI:** 10.1186/s12879-021-06401-3

**Published:** 2021-07-24

**Authors:** Magaly Lima Mota, Robson dos Santos Souza Marinho, Rodrigo Lopes Sanz Duro, James Hunter, Irwin Rose Alencar de Menezes, João Marcos Ferreira de Lima Silva, Glaubervânio Leite Tavares Pereira, Ester Cerdeira Sabino, Anete Grumach, Ricardo Sobhie Diaz, Maria do Socorro Lucena, Shirley Vasconcelos Komninakis

**Affiliations:** 1 Faculty of Medical of the ABC, Santo André, SP 09060-870 Brazil; 2 Centro Universitário Dr. Leão Sampaio, Juazeiro do Norte, CE 63040-405 Brazil; 3 Retrovirology Laboratory, Federal University of São Paulo, São Paulo, SP 04039-032 Brazil; 4 Universidade Regional do Cariri, Crato, CE 63105-010 Brazil; 5 Institute of Tropical Medicine, University of São Paulo, São Paulo, SP 05403-000 Brazil; 6 Central Public Health Laboratory, Juazeiro do Norte, CE 63010-464 Brazil

**Keywords:** Epidemiology, Dengue, Zika, Chikungunya, Brazil

## Abstract

**Background:**

The co-circulation of types of arbovirus in areas where they are endemic increased the risk of outbreaks and limited the diagnostic methods available. Here, we analyze the epidemiological profile of DENV, CHIKV and ZIKV at the serological and molecular level in patients with suspected infection with these arboviruses in the city of Juazeiro do Norte, Ceará, Brazil.

**Methods:**

In 2016, the Central Public Health Laboratory (LACEN) of Juazeiro do Norte received 182 plasma samples from patients who visited health facilities with symptoms compatible with arbovirus infection. The LACEN performed serological tests for detection of IgM/IgG to DENV and CHIKV. They then sent these samples to the Retrovirology Laboratory of the Federal University of São Paulo and Faculty of Medical of the ABC where molecular analyses to confirm the infection by DENV, ZIKV and CHIKV were performed. The prevalence of IgM/IgG antibodies and of infections confirmed by RT-qPCR were presented with 95% confidence interval.

**Results:**

In serologic analysis, 125 samples were positive for antibodies against CHIKV and all were positive for antibodies against DENV. A higher prevalence of IgG against CHIKV (63.20% with 95% CI: 45.76–70.56) than against DENV (95.05% with 95% CI: 78.09–98.12) was observed. When the samples were submitted to analysis by RT-qPCR, we observed the following prevalence: mono-infection by ZIKV of 19.23% (95% CI: 14.29–34.82) patients, mono-infection by CHIKV of 3.84% (95% CI: 2.01–5.44) and co-infection with ZIKV and CHIKV of 1.09% (95% CI: 0.89–4.56).

**Conclusion:**

The serologic and molecular tests performed in this study were effective in analyzing the epidemiological profile of DENV, CHIKV and ZIKV in patients with suspected infection by these arboviruses in the city of Juazeiro do Norte, Ceará/Brazil.

## Background

Dengue virus (DENV), Zika virus (ZIKV) and Chikungunya virus (CHIKV) are endemic arboviruses in tropical and subtropical regions of the world. Environmental changes and vector density, along with migration and immigration patterns of people contribute to the dissemination of these viruses and enable the occurrence of outbreaks or epidemics [1]. CHIKV belongs to the family Togaviridae (genus *Alphavirus*), DENV and ZIKV to the family Flaviviridae (genus *Flavivirus*). These viruses are transmitted to humans mainly by the mosquitoes *Aedes aegypti* and *Aedes albopictus*, although other forms of ZIKV transmission have been described [2–5].

In Brazil, DENV has circulated for over 30 years, with all of the four serotypes (DENV 1–4) causing a significant number of cases and several outbreaks in many Brazilian states [6]. The ZIKV virus was recently introduced in Brazil and the first report of transmission was in 2015 in the city of Natal in the state of Rio Grande do Norte. In 2014, the CHIKV had the first autochthonous cases confirmed in Oiapoque, in the state of the Amapá [7–9].

During the acute phase of infection, direct detection of the virus in the blood with RT-PCR (Reverse Transcriptase Polymerase Chain Reaction) is recommended. Still in this phase, antibodies of the class immunoglobulins M (IgM) can be easily detected by ELISA. The detection of immunoglobulins provides a wider window of opportunity for diagnosis because they remain detectable in the blood for longer periods of time [10, 11].

The presence of IgM and immunoglobulin G (IgG) should be interpreted carefully depending on the epidemiological context. During a typical primary infection with DENV, ZIKV and CHIKV, IgM is generally detectable between 3 and 14 days after infection. IgM reaches a peak and begins to decrease after approximately 2 weeks of illness IgM reaches a peak, which starts the production and detection of IgG [12].

Because many symptoms and clinical features of the infections by other arboviruses are similar to those of Dengue, the clinical diagnosis may be impaired. Additionally, the differential diagnosis among these infections by serology is often not possible due to the cross-reaction between the antibodies [13]. With the circulation of these three arboviruses, according to the Health Secretariat of the State of Ceará/Brazil, new epidemiological scenarios have been identified within of the state, which has increased the chance of occurrence of simultaneous infections, showing the need for differential diagnosis [14].

In Juazeiro do Norte, a municipality belonging to the Cariri metropolitan region in the State of Ceará, cases of infection by DENV, ZIKV and CHIKV are frequently reported precisely because it is considered one of the largest centers of popular religiosity in Latin America; which contributes for the movement of tourists in this region [15]; It is known that human behavior is an important determinant of arbovirus emergence. Currently, with displacement of people, adaptation and dispersion of mosquito vectors, cases of arbovirus infection have increased significantly [16]. Juazeiro do Norte is a city of many religious tourist attractions. Precisely because it is one of the largest cities in the state and because it receives so many visitors annually, it is difficult to control arboviruses in the city [17].

However, there is still little research that shows the epidemiological profile of these arboviruses at the serological and molecular level in this area. Our work had the objective of to analyze the epidemiological profile of DENV, CHIKV and ZIKV at the serological and molecular level in patients with suspected infection with these arboviruses in the city of Juazeiro do Norte, Ceará/Brazil.

## Methods

### Study design

In this cross-sectional observational study, we analyzed 182 plasma samples obtained from residents in Juazeiro do Norte. These individuals consulted different units of the public health system with symptoms suggestive of arbovirus infection. All these samples were collected in 2016. Based on the typical symptoms and epidemiology of the city, serological tests for DENV and CHIKV were requested for all patients. The Central Public Health Laboratory (LACEN) of Juazeiro do Norte received these samples and performed serological tests for detection of IgM or IgG to DENV and CHIKV. However, serological tests were not performed for ZIKV.

The samples were then sent to the Laboratory of Retrovirology of the Federal University of São Paulo and Medical School of the Federal University of ABC for molecular characterization of DENV, ZIKV and CHIKV by RT-qPCR. The serological and molecular analyses for this study were made between 3 and 14 days after the onset of symptoms, following criteria established by the World Health Organization (WHO) [18] and Centers for Disease Control and Prevention (CDC) [19]. The symptoms of the patients involved in this study were described by the original clinical units and sent to the LACEN. Afterwards, these data were also sent to the Retrovirology Laboratory and Faculty of Medical of the ABC.

### Study area

**Fig. 1:**
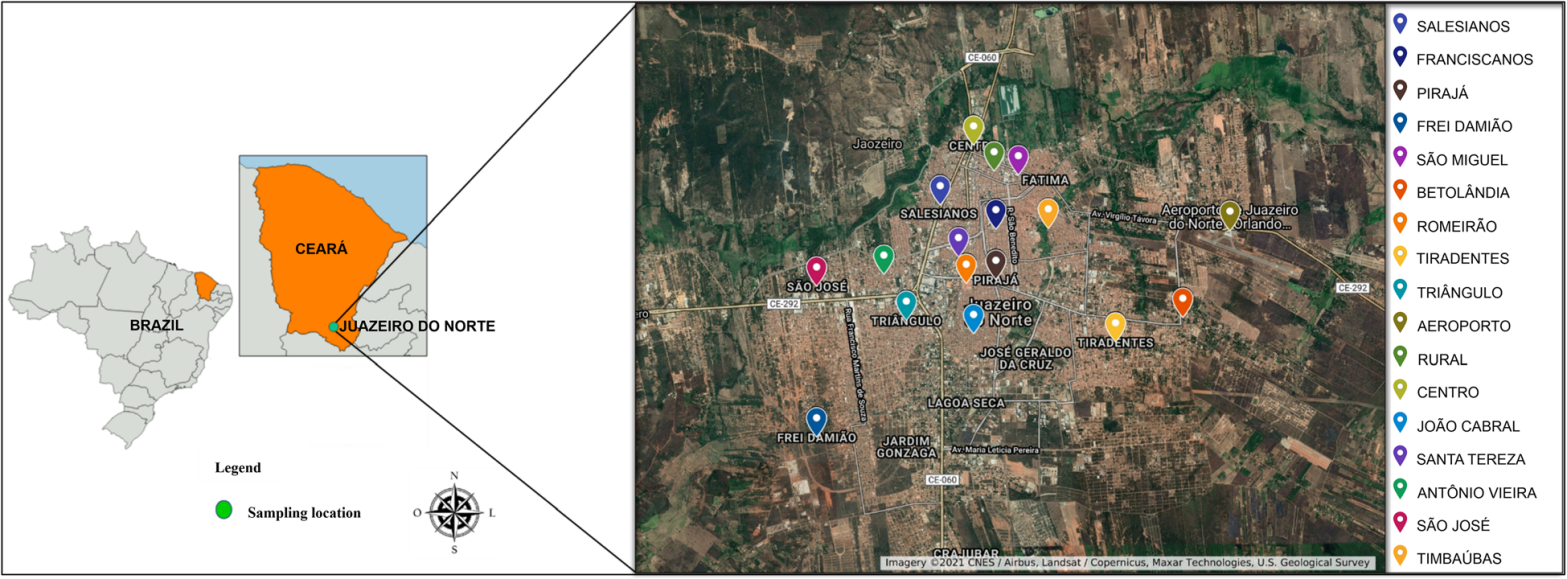
Location of the city of Juazeiro do Norte in state of the Ceará/Brazil and of the collection points of samples

Juazeiro do Norte is one of the nine municipalities that belong to the metropolitan region of Cariri, an urban complex located in the south of the state of Ceará (Fig. 1). According to the Brazilian Institute of Geography and Statistics (IBGE), it is the third most populous municipality in the state, with a territorial area of 248.6 km^2^, estimated population of 276, 264 people with a population density 1004.45 inhabitants/km^2^ and a human development index is 0.694 [20].

In 2016, the Secretary of Health of Ceará reported an outbreak of ZIKV and some cases of infection by CHIKV in the state with many cases in metropolitan region of Cariri [14]. All samples analyzed in this study were collected between January to October of 2016.

### Ethics statement and inclusion criteria

This study was approved by the Research Ethics Committee of the Federal University of Sao Paulo (CAAE: 57865416.4.0000.5505). All patients enrolled signed an informed written consent. The patients’ personal information was anonymized before the data was accessed. The information of the patients on demographic characteristics and clinical features were analyzed. This research involved people that were clinically diagnosed with some symptoms compatible with infection by arbovirus as fever, exanthema, headaches, myalgia, arthralgia, pruritus, and retro-orbital pain, more than 18 years of age, both sexes and resident in the municipality of Juazeiro do Norte.

### Methods statement

All methods described were performed in accordance with the relevant guidelines and regulations and were approved by the Research Ethics Committee of the Federal University of Sao Paulo.

### Serological analysis

All the plasma samples were tested for IgM and IgG by the method of Enzyme-Linked Immunosorbent Assay (ELISA). The detection of IgM/IgG against DENV and CHIKV was performed using the Euroimmun® kits (Lübeck, Schleswig-Holstein, Germany) following the manufacturer’s instructions. No serological test was requested for ZIKV. The results were interpreted according to the manufacturer’s instructions. Samples presenting inconclusive test results were retested to obtain a valid result.

### Molecular analysis

All samples analyzed in the serology were subjected to analysis by RT-qPCR. First, the viral RNA was extracted using the QIAamp Viral RNA Mini kit (Qiagen) following the manufacturer’s instructions. Posteriorly, ZDC multiplex real time RT-PCR assays (Bio-Rad) were performed. This kit simultaneously screens for the presence of Zika, Dengue and/or Chikungunya from a variety of sample types including serum, plasma, mosquito tissues, and larvae. It contains primers and probes as well as controls (positives and internal) needed for reliable results. The thermal profile consisted of a reverse transcription step at 50 °C for 30 min, activation of the enzyme at 95 °C for 5 min and 45 cycles of 95 °C for 15 s and 60 °C for 1 min for hybridization and extension. The reaction was realized in the ABI 7500 Real Time PCR system. All the procedures of this assays followed the manufacturer’s instructions.

### Statistical analysis

The percentage of women and men, the mean age of the patients and the mean of IgM/IgG and of Cycling threshold (Cts) were determined. The prevalence of antibodies (IgM and IgG) and of infections confirmed by RT-qPCR were presented with 95% confidence interval (95% CI). The statistical analyzes were performed using IBM SPSS® Statistics version 23.0.

## Results

### Serological characterization

Of the 182 samples, 125 were positive for antibodies against CHIKV and all were positive for antibodies against DENV. The 57 samples that were negative for CHIKV presented IgG positive for DENV. The serology showed a higher prevalence of IgG than IgM for CHIKV and DENV, as shown in Table 1. The positivity averages of IgM and IgG detected against CHIKV were 36.80% (SD ± 18.54) and 63.20% (SD ± 9.45) respectively. For DENV, IgM was 4.95% (SD ± 1.20) and IgG 95.05% (SD ± 28.35). Of the total number of patients involved in this study, 131 (71.98%) were women and 51 (28.02%) were men with the mean age of 40.06 years (SD ± 19.86).

**Table 1:** Detection of antibodies against CHIKV and DENV in the plasma samples of the patients attended at LACEN, Juazeiro do Norte, Ceará/Brazil

Arbovírus	Antibodies	Serology Results	N	% (95% CI)
CHIKV	IgM	Positive	46	36.80 (21.89–54.29)
	IgG	Positive	79	63.20 (45.76–70.56)
DENV	IgM	Positive	9	4.95 (1.92–8.34)
	IgG	Positive	173	95.05 (78.09–98.12)

*IgM* Immunoglobulin M, *IgG* Immunoglobulin G, *N* Number of individuals; *%* Prevalence, *CI* Confidence Interval

It was detected the presence simultaneous of antibodies IgM/IgG against the two arboviruses in the patients. All the 46 samples positive IgM for CHIKV were also positive IgG for DENV and in the 79 with positive IgG for CHIKV, 9 with positive IgM and 70 with positive IgG for DENV were verified.

### Molecular characterization

We observed a prevalence of 19.23% (95% CI: 14.29–34.82) patients confirmed with mono-infection by ZIKV, 3.84% (95% CI: 2.01–5.44) mono-infected by CHIKV and 1.09% (95% CI: 0.89–4.56) co-infected with ZIKV/CHIKV. The mean Cycling threshold (Cts) of the mono-infected patients by ZIKV was 28.90 Ct (SD ± 1.44) and by CHIKV was 27.42 Ct (SD ± 6.51). For the co-infecteds, it was 28.02 Ct (SD ± 3.93).

### Clinical characteristics of the patients confirmed by RT-PCR

We note that fever, exanthema, headaches, myalgia, arthralgia, and pruritus were the symptoms most reported by participants of this study. Fever, headaches, and arthralgia were observed in all patients with monoinfection by CHIKV and with coinfection by ZIKV/CHIKV. All clinical aspects of the patients confirmed with infections involving ZIKV and CHIKV are shown in the Table 2.

**Table 2:** Clinical features presented by the patients confirmed with monoinfection and coinfection with ZIKV and CHIKV by RT-qPCR

Clinical Features	Monoinfection and Coinfection (% Signs and Symptoms)		
	ZIKV (***n*** = 35)	CHIKV (***n*** = 7)	ZIKV/CHIKV (***n*** = 2)
Fever	29 (82.85)	7 (100)	2 (100)
Exanthema	27 (77.14)	6 (85.71)	2 (100)
Headaches	27 (77.14)	7 (100)	2 (100)
Arthralgia	19 (54.28)	7 (100)	2 (100)
Myalgia	25 (71.42)	4 (57.14)	2 (100)
Pruritus	19 (54.28)	1 (14.28)	0
Retro-orbital pain	13 (37.14)	2 (28.57)	1 (50)
Nausea	9 (25.71)	2 (28.57)	1 (50)
Vomiting	6 (17.14)	2 (28.57)	0

## Discussion

In the present study, we analyzed the serological and molecular epidemiology of DENV, ZIKV e CHIKV in city of Juazeiro do Norte in the state of the Ceará/Brazil. We observed that these viruses circulate concomitantly inside of the state mainly in the metropolitan Region of Cariri, which has increased the need for serological testing. However, these tests still has some limitations due to the cross reactions during the diagnosis of arboviruses. Thus, our study had the objective of contribute to this aspect helping to subsidize public policies that guarantee improvements epidemiological surveillance of arbovirosis in Brazil.

Our study detected high prevalence of IgG for DENV and CHIKV. According to the Secretary of Health of the State of Ceará [21], in 2014–2015, an increase in the number of cases of infection by DENV and CHIKV was registered. Juazeiro do Norte was one of the cities with large numbers of cases of infection by theses arbovirus in the state of Ceará. This may show that many participants in our study may have been exposed to Dengue or other flavivirus infection previously. Recently, a systematic review and meta-analysis performed by Li et al. [22] showed the worldwide seroprevalence of infection by DENV, CHIKV and ZIKV with the Southeast Asia Region having the highest seroprevalence of DENV and CHIKV. These seroprevalences were similar when comparing developed and developing countries, urban and rural areas or between different populations. These data reinforce our findings of high seroprevalence for these two arboviruses. Another aspect observed in our study were the samples that were positive for both IgM and IgG anti-DENV. The detection of these anti-DENV antibodies is widely used in the diagnosis of infection by DENV, as well as in sero-epidemiological investigations [23].

It is worth mentioning also that we observed 37 patients with serology positive for DENV (9 IgM and 28 IgG). However, during molecular characterization, we confirmed that they were positive for ZIKV but negative for DENV. Gouel-Cheron et al. [24] evaluated Zika diagnosis in symptomatic patients using serial RT-PCR and developed a classification model using serial Dengue virus (DENV) and ZIKV serologies. They noticed that diagnosis using serological tests in areas with co-circulating Flaviviruses may be inadequate due to cross-reactivities of Flavivirus-specific antibodies. They also demonstrated the importance of the RT-PCR to diagnose arbovirus infection in symptomatic patients living in regions with co-circulating flaviviruses. Therefore, establishing a final diagnosis of flavivirus in an endemic area of ZIKV/DENV co-circulation is a challenge, given the similarity of symptoms and the difficulty of serological tests to identify the viruses. New diagnostic criteria or algorithms are therefore necessary.

In this work, we observed 35 cases of patients monoinfected by ZIKV, confirmed by RT-qPCR, and the signs and symptoms most reported were fever (82.85%), exanthema (77.14%), headaches (77.14%), myalgia (71.42%), arthralgia (54.28%) and pruritus (54.28%). According to Brazil et al. [25], the clinical features commonly presented by patients infected with ZIKV during an outbreak that occurred in the state of Rio de Janeiro were exanthema (97%), followed by pruritus (79%), prostration (73%), headache (66%), arthralgia (63%) and myalgia (61%). A study conducted by Jimenez Corona et al. [26] analyzed 93 autochthonous cases of Zika in Mexico and observed that the main clinical characteristics presented by patients were fever (96.6%), exanthema (93.3%), headache (85.4%) and myalgia (84.3%). Thus, the clinical findings of patients characterized with monoinfection by ZIKV in our study corroborate with some studies previously performed.

Different than ZIKV, where most infections are asymptomatic, CHIKV infections are usually symptomatic. In our study, we observed 7 cases of mono-infection by CHIKV. All these patients reported arthralgia, fever and headache. Exanthema (85.71%) and myalgia (57.14%) were also frequently reported. According to Silva et al. [27], a characteristic clinical feature of the infection by CHIKV is arthralgia/polyarthralgia, which is present in approximately 50–97% of individuals infected with this virus. Moreover, a cross-sectional study realized in an urban slum in Brazil to determine the local rate of transmission of CHIKV showed that other clinical manifestations in addition to arthralgia are compatible with mono-infection by CHIKV, such as fever, exanthema, myalgia, headache, among others [28]. This corroborates the clinical aspects observed in patients positive for CHIKV in our study.

Another important finding in our study was the detection of 2 cases of co-infection between ZIKV and CHIKV. Some studies show that double infection involving these two arboviruses can cause more severe clinical conditions [29]. However, we did not observe severe symptoms in the patients detected with this coinfection. We noticed that fever, exanthema, headache, arthralgia, and myalgia were common symptoms for both patients. Zambrano et al. [30] described three cases of coinfection with ZIKV/CHIKV in Ecuador. They pointed out that the clinical characteristics presented by co-infected patients in non-severe cases are similar to those of mono-infected patients with one of these two viruses. This supports our clinical findings for this group of patients analyzed.

The co-circulation of different arboviruses in Brazil presents a major challenge to national public health policy in terms of diagnosis and the confirmation of cases. However, our study has some limitations due to the low number of clinical samples that we received for analysis. As this was a retrospective study, it was not possible to obtain complete medical information from the participants. In addition, serological analyzes for ZIKV that were not performed by LACEN and the Laboratory of Retrovirology and the Faculty of Medical of the ABC were not this analysis because the samples did not have sufficient volume.

This work reinforced that there is simultaneous circulation of different arboviruses in the municipality of Juazeiro do Norte/Ceará. However, studies of serological and molecular epidemiology need to be performed to confirm the possible circulation of other arboviruses (not DENV, CHIKV and ZIKV) and the detection of co-infection among them in the state of Ceará. The Mayaro virus is present in the states of Mato Grosso, Goiás and Rio de Janeiro. There are records of cases of yellow fever in the states of Bahia and Rio Grande do Norte. Finally, the Oropouche virus has been shown to circulate in the northeast region of Brazil. This indicates that more serological and molecular studies of arbovirus infection need to be performed in Brazil, especially in metropolitan region of Cariri in Ceará.

## Conclusion

From the data obtained by this study, it can infer that serological methods should not be the only analysis tool in the diagnosis of arboviruses in areas where they are endemic. The absence of molecular tests in the year 2016 led to errors in diagnosis of ZIKV, CHIKV and DENV in Juazeiro do Norte, one of the main cities of the State of Ceará. Thus, the combination of the serological and molecular analyzes carried out in this study were effective in the evaluation of the epidemiological profile of DENV, CHIKV and ZIKV in patients with suspected infection with these arboviruses in the city of Juazeiro do Norte, Ceará. Thus, our findings reinforce the need for further studies of serological and molecular tests to differentiate and confirm arbovirus infections.

## Data Availability

Please contact the corresponding author for data requests.

## Abbreviations

CHIKV: Chikungunya Virus
DENV: Dengue Virus
WHO: World Health Organization
ZIKV: Zika Virus
RT-qPCR: Quantitative Reverse transcription polymerase chain reaction
CDC: Centers for Disease Control and Prevention
CI: Confidence Interval
LACEN: Central Public Health Laboratory
ELISA: Enzyme-Linked Immunosorbent Assay
IgM: Immunoglobulin M
IgG: Immunoglobulin G
Ct: Cycling threshold
